# Risk Factors of Postoperative Acute Heart Failure in Elderly Patients After Hip Fracture Surgery

**DOI:** 10.7759/cureus.58967

**Published:** 2024-04-25

**Authors:** Muhammad Hamza Riaz, Javaria Riaz, Asim Mahmood, Manahil Tariq, Nabiha Sahar, Rana Shahzaib Ali, Nadeem Ahmad, Sumbal Irshad, Muhammad Hassan Ahmad, Hamid Arshad, Tayyab Mumtaz Khan

**Affiliations:** 1 Cardiology, Allama Iqbal Medical College, Lahore, PAK; 2 Medicine, Mohi-ud-Din Islamic Medical College, Mirpur, PAK; 3 Cardiology, Lahore General Hospital, Lahore, PAK; 4 Cardiology, Jinnah Hospital, Lahore, PAK; 5 Internal Medicine, Allama Iqbal Medical College, Lahore, PAK; 6 Orthopedic Surgery, Sheikh Zayed Medical College and Hospital, Rahim Yar Khan, PAK; 7 Internal Medicine, Philadelphia College of Osteopathic Medicine, Philadelphia, USA; 8 Medicine, Allama Iqbal Medical College, Lahore, PAK; 9 Surgery, Allama Iqbal Medical College, Lahore, PAK; 10 Orthopedic Surgery, Rawalpindi Medical University, Rawalpindi, PAK

**Keywords:** surgery, fracture, hip, elderly, postoperative, acute heart failure, risk factor

## Abstract

Background

Postoperative acute heart failure (AHF) in elderly patients after hip fracture surgery is a common complication. Therefore, this study aimed to identify the risk factor of AHF after hip fracture surgery among the older population.

Methods

This retrospective cohort study was performed on 88 admitted patients whose hip fractures were fixed via internal fixation surgery in a tertiary care hospital in Rawalpindi, Pakistan, from January 2022 to March 2023. Recruitment of patients was made through established inclusion and exclusion criteria. Ethical approval and informed consent were also gained before the data collection. A self-designed form was used to collect data. Data analysis was carried out in the IBM SPSS Statistics for Windows, Version 25 (Released 2017; IBM Corp., Armonk, New York, United States). Both descriptive and inferential statistics were applied to compare the attributes of the patients with AHF and patients without AHF. Multivariate logistic regression was used to evaluate the association between the postoperative AHF and its potential risk factors.

Results

Out of 88 enrolled patients, 12 (13.64%) had developed postoperative AHF. Age ≥ 65 years (OR = 2.606, 95% CI = 1.035~4.160, p = 0.010), anemia (OR = 3.178, 95% CI = 1.847~5.990, p = 0.029), hypertension (OR = 2.019, 95% CI = 1.110~4.034, p = 0.012), diabetes mellitus (OR = 2.003, 95% CI = 1.115~4.012, p = 0.015), hypoalbuminemia (OR = 2.486, 95% CI = 1.218~4.619, p = 0.030), and operation time ≥ 120 minutes (OR = 1.702, 95% CI = 1.099~2.880, p = 0.018), were the risk factors of postoperative AHF in elderly patients after hip fracture surgery.

Conclusions

In the study population, the incidence of postoperative heart failure was significant and age ≥ 65 years, anemia, hypertension, diabetes mellitus, hypoalbuminemia, and operation time ≥ 120 were significantly involved in the development of it. Preoperative identification and management of AHF risk factors could lead to the prevention of postoperative complications.

## Introduction

The worldwide incidence of hip fractures in the elderly is high. It is estimated that the number of cases of hip fractures will be 6.3 million in 2050 [1]. Currently, the United States (US) has one of the highest rates of hip fractures worldwide, with an annual incidence of over 340,000 hip fractures [2]. While in China, it is expected that the annual number of hip fractures will cross 500,000 by 2040 [3]. Age-related medical conditions such as osteoporosis and fragility fractures are the main causes that lead to hip fractures, even after minor trauma. As the elderly population is increasing globally, it is also anticipated that the burden of hip fractures will double in the next 5-10 years [2,4].

Main hip fracture types include neck of femur fracture, intertrochanteric fracture, subtrochanteric fracture, and proximal shaft of femur fracture. Surgery is the main modality of treatment for hip fractures that need hospitalization and subsequent rehabilitation [5]. One of the major causes of poor quality and disability in older people is hip fracture. Moreover, studies have also shown that the risk of death is higher among elderly people with hip fractures in contrast to the population without fractures. Postoperative complications which are common after hip fracture surgery among the elderly could be the causes of a higher rate of death in the older population [6]. After hip fracture surgery, the rates of postoperative complications range from 12.5% to 40.0% [1]. The mortality rate is approximately 4.5-10.0% during the acute period, which is the first month following the injury, and it is almost 14-36% within the first year of the injury [7,8].

One of the most common and fatal postoperative complications among the elderly after hip fracture surgery is acute heart failure (AHF) [9]. The rate of postoperative AHF ranges from 6% to 20% in the elderly after hip fracture surgery [10]. AHF may result in slow recuperation, prolonged hospital stays, higher medical expenses, additional issues like pressure sores, and consequent major negative impacts on the patient's quality of life and ability to function physically [4,11]. Hip fractures in the US are thought to cost over $12 billion annually on average, placing a heavy financial strain on the healthcare system [8].

Identification of the risk factors that cause postoperative AHF could help in reducing the morbidity and mortality after hip fracture surgery in elderly patients and the subsequent financial burden on the healthcare system. Therefore, this study aims to explore the potential risk factors of AHF in elderly patients after hip fracture surgery.

## Materials and methods

Study design and study population

This retrospective cohort study was conducted in the Orthopedics department of Benazir Bhutto Hospital, Rawalpindi, Pakistan. Data from 88 patients whose hip fractures were managed via internal fixation surgery from January 2022 to March 2023 were collected from medical records.

Inclusion and exclusion criteria

All included patients were aged 55 years or above and their hip fractures were fixed through surgery. Whereas, patients with a previous history of heart disease, incomplete data, old and pathological fractures or multiple fractures, and pulmonary embolism prior to surgery were excluded from the study.

Acute heart failure diagnosis

To find out if patients had postoperative AHF, the researchers in this retrospective analysis went through the patient's medical data. The European Society of Cardiology's 2021 guidelines for the diagnosis and management of acute and chronic heart failure were cited in the diagnostic criteria for AHF. The diagnosis of AHF was made on the basis of clinical features such as dyspnea, rapid heartbeat, third heart sound, raised jugular vein pressure, lung rales, and lower limb edema. In addition, elevated B-type natriuretic peptide (BNP) level was also taken into account. Patients were divided into two groups based on the presence of heart failure.

Ethical approval

Ethical approval was acquired from the Ethical Review Board (ERB) of Benazir Bhutto Hospital, Rawalpindi, Pakistan. The ethical approval number was BBH.ERB.283/169. Informed consent was also obtained.

Data collection

A self-structured form was used for data collection. It had three parts. The first part included sociodemographic variables such as age (less than 65 years and 65 and above years), gender (male or female), body mass index (BMI), fracture type (femoral neck fracture or intertrochanteric fracture), and comorbidities presence or absence (anemia, hypertension, diabetes mellitus, hypoalbuminemia hyperlipidemia, and impaired cognition). The second part was about variables linked with operation like anesthesia type (spinal or general), duration of operation (less than 120 minutes or 120 minutes and above), and approximate blood loss during operation (less than 200 ml or 200 ml and above). The third part comprised biochemical parameters hemoglobin, serum albumin level, serum lipid level, and BNP. BMI was measured by dividing the weight by height squared. Blood loss was calculated by following the formula blood loss = (blood gauze weight - dry gauze weight) + suction bottle blood volume).

Data analysis

The statistical analysis in this study was performed using IBM SPSS Statistics for Windows, Version 25 (Released 2017; IBM Corp., Armonk, New York, United States). The numerical data were presented as mean ± standard deviation while the nominal data were shown as percentages. Independent samples t-tests and chi-square tests were used to compare the groups for quantitative and qualitative variables, respectively. AHF risk factors were examined in them by using a multivariate logistic regression model. In this study, p < 0.0 5 was set as statistically significant.

## Results

Of the total of 88 patients, 12 (13.64%) patients had AHF after hip fracture surgery.

Table 1 indicates the characteristics of the study participants. Statistically significant differences were noted between the AHF group and the non-AHF group, in age, anemia, hypertension, diabetes mellitus, hypoalbuminemia, and duration of operation with p ≤ 0.05 for all these factors. Whereas, there were no significant differences between the AHF and non-AHF groups, in gender, BMI, hyperlipidemia, cognitive impairment, fracture type, blood loss in operation, and anesthesia type with p ≥ 0.05 for all these variables.

**Table 1:** Characteristics of the study population along with the independent t-test and chi-square test analysis

Variables			Acute heart failure group (n = 12)	Non-acute heart failure group (n = 76)	Independent t-test/chi-square test (p-value)
Age (years)			72.23 ± 8.44	62.70 ± 6.05	0.039
Gender		Male	7 (58.33%)	40 (52.63%)	0.080
		Female	5 (41.67%)	36 (47.37%)	
BMI (kg/m^2^)			23.24 ± 4.32	22.90 ± 5.76	0.091
Anemia			8 (66.67%)	18 (23.68%)	0.022
Hypertension			9 (75.0%)	24 (31.57%)	0.005
Diabetes mellitus			4 (33.33%)	19 (25.0%)	0.045
Hypoalbuminemia			4 (33.33%)	12 (15.78%)	0.009
Hyperlipidemia			3 (25.0%)	17 (22.36%)	0.108
Cognitive impairment			1 (8.33%)	6 (7.89%)	0.078
Fracture type	Intertrochanteric fracture		5 (41.67%)	32 (42.10%)	0.120
	Neck of femur fracture		7 (58.33%)	44 (57.90%)	
Operation time (minutes)			140.23 ± 59.12	111.09 ± 62.23	0.003
Blood loss in operation (ml)			246.12 ± 58.09	239.43 ± 52.67	0.069
Anesthesia type	Spinal		5 (41.67%)	35 (46.05%)	0.100
	General		7 (58.33%)	41 (53.95%)	

Table 2 shows the variables allotments of multivariate logistic regression. Factors with significant difference in the univariate analysis were added in multivariate logistic regression for further evaluation.

**Table 2:** Allotment of variables of multivariate logistic regression

Factors	Allotment	Variables
Heart failure	Y	1 = Yes, 2 = No
Age ≥ 65 years	X1	1 ≥ 65, 2 ≤ 65
Anemia	X2	1 = Yes, 2 = No
Hypertension	X3	1 = Yes, 2 = No
Diabetes mellitus	X4	1 = Yes, 2 = No
Hypoalbuminemia	X5	1 = Yes, 2 = No
Operation time ≥ 120 minutes	X6	1 ≥ 120, 2 ≤ 120

Table 3 manifests that logistic regression analyses showed that age ≥ 65 years (OR = 2.606, 95% CI = 1.035~4.160, p = 0.010), anemia (OR = 3.178, 95% CI = 1.847~5.990, p = 0.029), hypertension (OR = 2.019, 95% CI = 1.110~4.034, p = 0.012), diabetes mellitus (OR = 2.003, 95% CI = 1.115~4.012, p = 0.015), hypoalbuminemia (OR = 2.486, 95% CI = 1.218~4.619, p = 0.030), and operation time ≥ 120 minutes (OR = 1.702, 95% CI = 1.099~2.880, p = 0.018), were the risk factors of postoperative AHF in elderly patients after hip fracture surgery.

**Table 3:** Logistic regression analysis on the risk factors of postoperative heart failure OR: odds ratio; Cl: confidence interval

Variables	Beta coefficient	OR	95% CI	p-value
Age ≥ 65 years	0.108	2.606	1.035~4.160	0.010
Anemia	0.141	3.178	1.847~5.990	0.029
Hypertension	0.182	2.019	1.110~4.034	0.012
Diabetes mellitus	0.178	2.003	1.115~4.012	0.015
Hypoalbuminemia	0.152	2.486	1.218~4.619	0.030
Operation time ≥ 120 minutes	0.130	1.702	1.099~2.880	0.018

## Discussion

The risk of hip fractures goes up with the aging of the population and surgery is the main approach to hip fracture management. However, the presence of comorbidities and age-related decline in body organ functions lead to many postoperative complications in elderly people after hip fracture surgery. One of the most significant and frequent complications is postoperative AHF. In view of the significant incidence of postoperative AHF in the elderly, determining the potential risk factors of AHF after hip fracture surgery could help physicians in controlling it by identifying the most vulnerable patients and preoperative management of possible risk factors.

In this study, we observed that the incidence of postoperative AHF in the elderly after hip fracture surgery was 13.64%. Almost similar incidence of postoperative AHF (12.37%) was also noted in a study in China [11]. Another study reported a higher incidence of AHF (18.37%) after hip fracture surgery [9]. In literature has shown that the overall incidence of cardiovascular events after hip surgery in the elderly could be up to 40% [1]. These variations in the percentages of cardiovascular complications in various regions of the world could be due to differences in the prevalence of different potential risk factors of the AHF.

Aging leads to raised hip fracture frequency and subsequent increased incidence of postoperative AHF as well. Another study has endorsed the present study's idea that old age itself is a risk factor for AHF after hip fracture surgery [12]. The mechanism through which aging leads to increased AHF postoperatively is the structural and functional deterioration of the heart with aging [13]. As aging is a non-modifiable risk factor, therefore, by preventing the other co-exited and modifiable risk factors, we can reduce AHF frequency in elderly people who need hip surgery.

Anemia was also a risk factor for postoperative AHF in the elderly who had undergone hip fracture surgery. A study that was carried out in different parts of the world has also supported this finding of our study [14]. Anemia causes hypoxia of tissues, subsequent vasodilation, and reduced peripheral resistance, which leads to stimulation of the renin-angiotensin system and adrenal sympathetic system, which ultimately retain water and sodium, resulting in heart failure [15].

Another significant risk factor of postoperative AHF in the study population was hypertension which increases the workload on the heart. In literature, various studies have backed this association between AHF and hypertension in patients who have undergone hip repair surgery [8,13].

In this study population diabetes mellitus was also an important risk factor for the development of postoperative AHF. We found consistent findings regarding the impact of diabetes mellitus in different studies around the globe [1,6].

In addition, it was observed in the results of the present study that adequate albumin level is essential for the prevention of postoperative complications in the elderly as hypoalbuminemia which represents the malnutrition state of the body, was linked with postoperative AHF significantly. In previous studies, this correlation between postoperative AHF and hypoalbuminemia was also noted worldwide [2,10].

Regarding the connection between acute cardiac failure and hip surgery duration, this study discovered that the risk of AHF increased with the increase in the length of the procedure. One possible explanation is that the patient experiences more stress and subsequently the heart remains under higher strain during the longer duration of the procedure. Furthermore, during surgery, patients frequently receive a specific amount of fluid infusion; the longer the operation, the more fluid will be infused, and the patient's cardiac load will rise noticeably. Both these mechanisms collectively result in acute cardiac events. Alike influence of the operation time on acute cardiac events was also affirmed in another research article [11].

The merit of this study is that according to our knowledge, this study is the only one that has highlighted the potential risk factors of postoperative AHF in the elderly population after hip fracture surgery. However, this study has also some limitations. First, this was a single-center study which could be the cause of a bias. Second, it had a small sample size. Therefore, this study also encourages other researchers to conduct future studies in multiple centers and with large sample sizes to avoid any kind of bias.

## Conclusions

This current study has shown that the incidence of postoperative AHF in older patients who have undergone hip fracture surgery was relatively high. It has also been demonstrated that age ≥ 65 years, anemia, hypertension, diabetes mellitus, hypoalbuminemia, and operation time ≥ 120 minutes were the significant risk factors for postoperative AHF in the elderly population. Therefore, this study recommends that in clinical practice, early detection and correction of hypertension, anemia, and hypoalbuminemia prior to surgery, as well as a reduction in the length of the surgical procedure, are necessary to lower the incidence of postoperative AHF.

Proper assessment and forecasting enable patients to reduce the potential risks of surgery, effectively beat the postoperative challenges, and maximize their degree of recovery.
